# Comorbidity in lung cancer patients and its association with medical service cost and treatment choice in China

**DOI:** 10.1186/s12885-020-06759-8

**Published:** 2020-03-24

**Authors:** Ruoxi Ding, Dawei Zhu, Ping He, Yong Ma, Zhishui Chen, Xuefeng Shi

**Affiliations:** 1 Institute of Population Research, Peking University, Beijing, 100871 China; 2 China Center for Health Development Studies, Peking University, No. 38 Xueyuan Road, Haidian District, Beijing, 100191 People’s Republic of China; 3 China Health Insurance Research Association, Beijing, 100013 China; 4 Department of Medical Insurance, Peking University Cancer Hospital and Institute, Key Laboratory of Carcinogenesis and Translational Research (Ministry of Education), Beijing, 100142 China; 5 School of Management, Beijing University of Chinese Medicine, No. 11, Bei San Huan Dong Lu, Chaoyang District, Beijing, 100029 China

**Keywords:** Medical service, Cost, Treatment choice, Comorbidity, Lung cancer, China

## Abstract

**Background:**

It is evident that comorbidity exacerbate the complexity of the management of lung cancer, however, limited research has been conducted to investigate the impact of comorbidity on health service utilization and cost, as well as the treatment choice among lung cancer patients. We examined the association of comorbidity with medical service utilization, cost and treatment choice among lung cancer patients in China.

**Methods:**

We used claims data from China Urban Employees’ Basic Medical Insurance (UEBMI) and Urban Residents’ Basic Medical Insurance (URBMI) between 2013 to 2016 and data from Hospital Information System (HIS) Database in Beijing Cancer Hospital (BCH). Elixhauser Comorbidity Index was used to assess comorbidity. Negative binomial regression, generalized linear model (GLM) with a gamma distribution and a log link, and logistic regression was applied to assess the associations between comorbidity and medical service utilization, cost and treatment choice, respectively.

**Results:**

Among 8655 patients with lung cancer, 31.3% of had at least one comorbid conditions. Having comorbidity was associated with increased number of annual outpatient visits (1.6, 95%CI: 1.3, 1.9) and inpatients admissions (0.8, 95%CI, 0.70, 0.90), increased outpatient (USD635.5, 95%CI: 490.3, 780.8) and inpatient expenditure (USD2 470.3, 95CI%: 1998.6, 2941.9), as well as increased possibility of choosing radio therapy (OR: 1.208, 95%CI:1.012–1.441) and chemotherapy (1.363, 1.196–1.554), and decreased possibility of choosing surgery (0.850, 0.730–0.989). The medical utilization and expenditure, the possibility of choosing radiotherapy increases, and the possibility of choosing surgery decreases with the increasing number of chronic conditions. There are variations in the association with medical service utilization and expenditure, and treatment choice among individuals with different types of comorbid conditions.

**Conclusion:**

Comorbidity among lung cancer patients restricts the potential treatment choices and poses an extra substantial health care burden. Our findings provide implications for both the clinical management and health service planning and financing for lung cancer patients.

## Background

Lung cancer remains one of the leading cause of cancer incidence and mortality throughout the world and it has imposed a substantial disease burden to global public health [1]. Lung cancer is one of the main contributors to cancer-caused disability adjusted life years (DALYs) in most nations [2] and patients diagnosed with lung cancer always experienced significant cost and medical service utilization. In United States, lung cancer accounts for approximately 20% of Medicare’s total cancer treatments expenditure [3]. With the growing incidence and mortality rate, China has experienced a significant increase in the relative disease burden of lung cancer, with 12% of total DALYs from cancers in 1990 to 20% in 2008 [2]. In 2015, the total direct medical cost of lung cancer was estimated to be $ 3.9 billion, and the average annual medical expenditure per patient ranges from $ 8522 to $ 14,519 [4], accounted for more than 150% of household annual income in 2015 [5]. China faces up with severe predicament of lung cancer burden. According to the prediction of The World Health Organization, the annual number of new cases of lung cancer death in China will be over one million by 2025 [6], and the expenditure will become even more burdensome to the entire society as the rapid population aging.

Majority of patients with lung cancer are diagnosed at advanced age and always comorbid other chronic diseases. The prevalence of comorbidity among lung cancer patients ranges from 43.3% in Sweden [7] and 87.3% in Scotland [8]. Comorbidity has been associated with delayed diagnosis and deteriorated performance status in patients with lung cancer. And low use of curative-intent or aggressive therapy has been reported among cancer patient with comorbidity due to treatment-related toxicity and adverse quality of life effects. It has also been suggested that comorbidity in general population may increase the therapeutic difficulty, and further incur extra medical service utilization and expenditure. Results from French citizen revealed that non-communicable diseases multi-morbidity is associated with greater primary and secondary healthcare utilization and economic burden [9]. In China, the average expenditure of outpatient serviced increased from 131.6 Chinese Yuan in patients with single chronic diseases to 179.2 in patients had multiple non-communicable diseases [10]. It is evident that comorbidity exacerbate the complexity of the management of lung cancer. However, limited research has been conducted to investigate the impact of comorbidity on health service utilization and cost, as well as the treatment choice among lung cancer patients, neither in developed countries nor in those with a rapid increasing rate of incidence, such as China.

In this study, we combined claims data from China Urban Employees’ Basic Medical Insurance (UEBMI) and China Urban Resident’s Basic Medical Insurance (URBMI), which covered more than 93% of the residents in urban China, and data from Hospital Information System (HIS) Database in Beijing Cancer Hospital (BCH), to examine the association of the presence, the number and the different types of comorbidity with 1. medical service utilization, 2. medical service cost and 3. treatment choice among lung cancer patients. It will provide implications for both the clinical management and health service planning and financing for lung cancer patients.

## Methods

### Data source

Currently there is no single database in China could provide the information on all the main variables (comorbidity, medical service utilization, medical service cost and received treatment) in our study. Therefore, we combined two data set---- claims data from Urban Employees’ Basic Medical Insurance (UEBMI) and Urban Residents’ Basic Medical Insurance (URBMI), and data from Hospital Information System (HIS) Database in Beijing Cancer Hospital (BCH) to examine the association between comorbidity and medical service utilization, cost and treatment choice among lung cancer patients.

In the first part analysis, we used claims data from Urban Employees’ Basic Medical Insurance (UEBMI) and Urban Residents’ Basic Medical Insurance (URBMI) between 2013 to 2016 in the first part analyses. Those two insurances covered more than 93% of the residents in the urban China in 2016 [11]. The data was collected by the China Health Insurance Research Association (CHIRA), and contain all the records of urban population’s demographic information and diagnoses of hospital admissions and outpatient visits. Based on the availability of other diagnosis and geographic location, seven cities (Beijing, Hangzhou, Fuzhou, Hefei, Changsha, Chengdu, and Kunming) were selected, and a 2% random sample of all beneficiaries were chosen by systematic sampling.

In the second part analyses, we collected additional data from Hospital Information System (HIS) Database in Beijing Cancer Hospital (BCH) from January 2016 to March 2018. Beijing University Cancer Hospital was established in 1976, and it has been one of the top cancer hospitals in China. It has 1040 health professionals and 790 hospital beds, with 450 thousand outpatient visits and 40 thousand inpatients admissions in 2013. The HIS database contains diagnostic and basic socio-demographic information.

### Measurements

#### Lung cancer

In both first and second part analysis, ICD-10 (the 10th revision of the International Statistical Classification of Diseases) was used to identify patients with lung cancer based on principal diagnosis codes C34. Diagnosis of lung cancer was made by qualified clinical practitioners according to Chinese Guidelines on the Diagnosis and Treatment of Primary Lung Cancer (2011 version to 2016 version). The cardinal diagnosis included strictly and comprehensive general clinical examination, such as endoscopy and pathological test.

#### Comorbidity

According to the comorbidity characteristics of our sample, we use Elixhauser Comorbidity Index with ICD-10 coding algorithms [12] to assess comorbidity in both first and second part analysis. Two variables were used to measure the condition of comorbidity: whether having comorbid conditions and the number of chronic conditions, and we divided the number of chronic conditions into four categories: 0, 1, 2 and ≥ 3. To examine the association of the different types of comorbidity with medical service utilization and expenditure among lung cancer patients, comorbid conditions were classified into six main types of comorbidity: Other malignancy (lymphoma, metastatic cancer, solid tumor without metastasis), hypertension (uncomplicated and complicated), pulmonary disease (pulmonary circulation disorders, chronic pulmonary disease), diabetes mellitus (uncomplicated and complicated), cardiovascular disease (congestive heart failure, cardiac arrhythmias, vascular disease), and liver disease.

#### Medical service utilization and expenditure

In the first part analysis, the information on medical service utilization and expenditure were extracted based on the condition that lung cancer was claimed as the index disease. Medical service utilization included the number of annual outpatient visits and the number of annual inpatient admissions. Medical service expenditure was measured by the annual outpatient expenditure, annual inpatient expenditure, the annual outpatient out-of-pocket (OOP) expenditure, and the annual inpatient OOP expenditure in RMB (In China, patients were asked to pay all types of medical fee immediately every time. The OOP expenditure will be paid by patients themselves, and the part that covered by medical insurance will be settled with hospitals at the end of every year. The annual total and OOP expenditure was clearly recorded in the claims data). The expenditure in US Dollars was also calculated based on the RMB against USD exchange rate of 0.159 (the average annual exchange rate from 2013 to 2016) [13]. All the expenditure included spending on pharmacy, diagnostic tests, and medical service fee.

#### Treatment

In the second part analysis, four types of treatment were identified: targeted therapy, radiotherapy, chemotherapy and surgery. Considering the fact that most of patients who received surgery also received other treatment like chemotherapy or radiotherapy, targeted therapy/radiotherapy/ chemotherapy was defined as patients received targeted therapy/radiotherapy/ chemotherapy and never received surgery. Surgery was defined as patients ever received surgery, irrespective of the use of other treatment.

#### Control variables

In both the first and second part analysis, control variables included age groups (younger than 60 years, 60–69 years, and 70 years or older), gender (male and female), insurance type (UEBMI and URBMI), lung cancer stage (I, II, IIIA, IIIB and IV) (only in the second part analysis), which was defined by Tumor-Node-Metastasis (TNM) classification, and year (2013, 2014, 2015 and 2016).

### Ethical approval

Since the data sets we used were anonymized database and had no impact on patients’ health and care, the informed consent was exempted. This study was approved by the Ethics Committee of Beijing University of Chinese medicine (No.2019BZHYLL0201).

### Statistical analysis

Descriptive analysis was used to analyze the sample characteristics in both first and second part analysis. In the first part analysis, associations between medical service utilizations (outpatient visits and inpatient admissions) and the presence of comorbidity, the number of comorbidity and the type of comorbidity were evaluated by negative binomial regression since over-dispersion is present. Generalized linear model (GLM) with a gamma distribution and a log link was used to assess the association of annual total and out-of-pocket (OOP) medical care expenditure with the presence, the number and the type of comorbidities. In the second part analysis, logistic regression was used to evaluate the contribution of the presence, the number and different types of comorbidity to different type of treatment choice (targeted therapy, radiotherapy, chemotherapy and surgery).

A *p*-value of less than 0.05 was considered statistically significant. The software Stata version 15 for Windows (Stata Corp, College Station, TX, USA) was used for the statistical analysis.

## Result

### Sample characteristics

In the first part analysis, 8655 patients with lung cancer were identified from the claims data between 2013 and 2016. The median age was 65 years, 60% of patients were male, and 80.9% of the sample was covered by UEMBI (Table 1). In the second part analysis, 5338 patients with lung cancer were identified from HIS Database in Beijing Cancer Hospital (BCH) from 2016 to 2018. The median age was 60 years, 61.3% of patients were male, and 68.6% of patients were covered by UEBMI (Table 2).

**Table 1:**
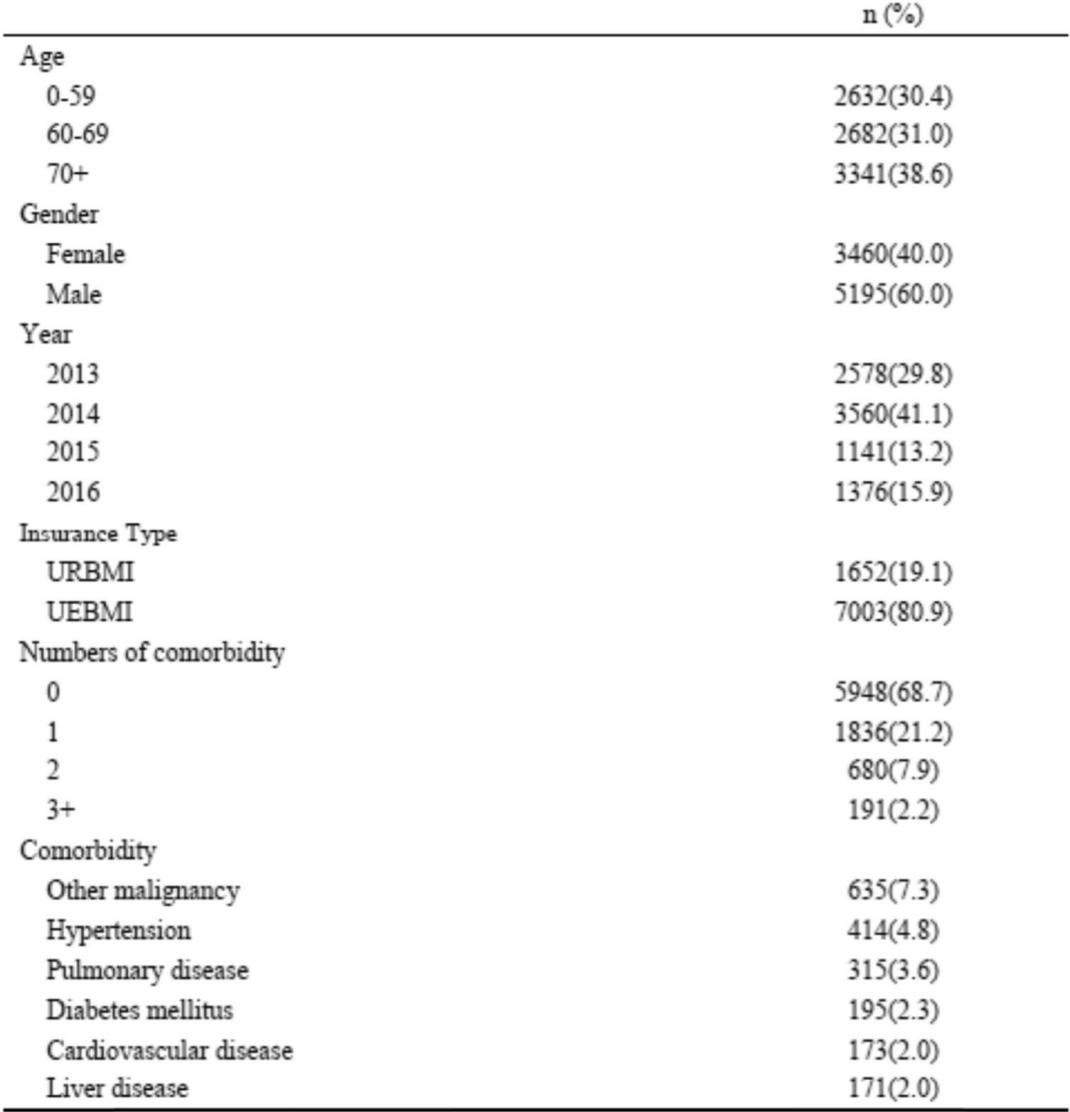
Sample characteristics (*n* = 8655)^1^

^1^First part analysis: claims data

*URBMI* Urban Residents’ Basic Medical Insurance, *UEBMI* Urban Employees’ Basic Medical insurance

**Table 2:**
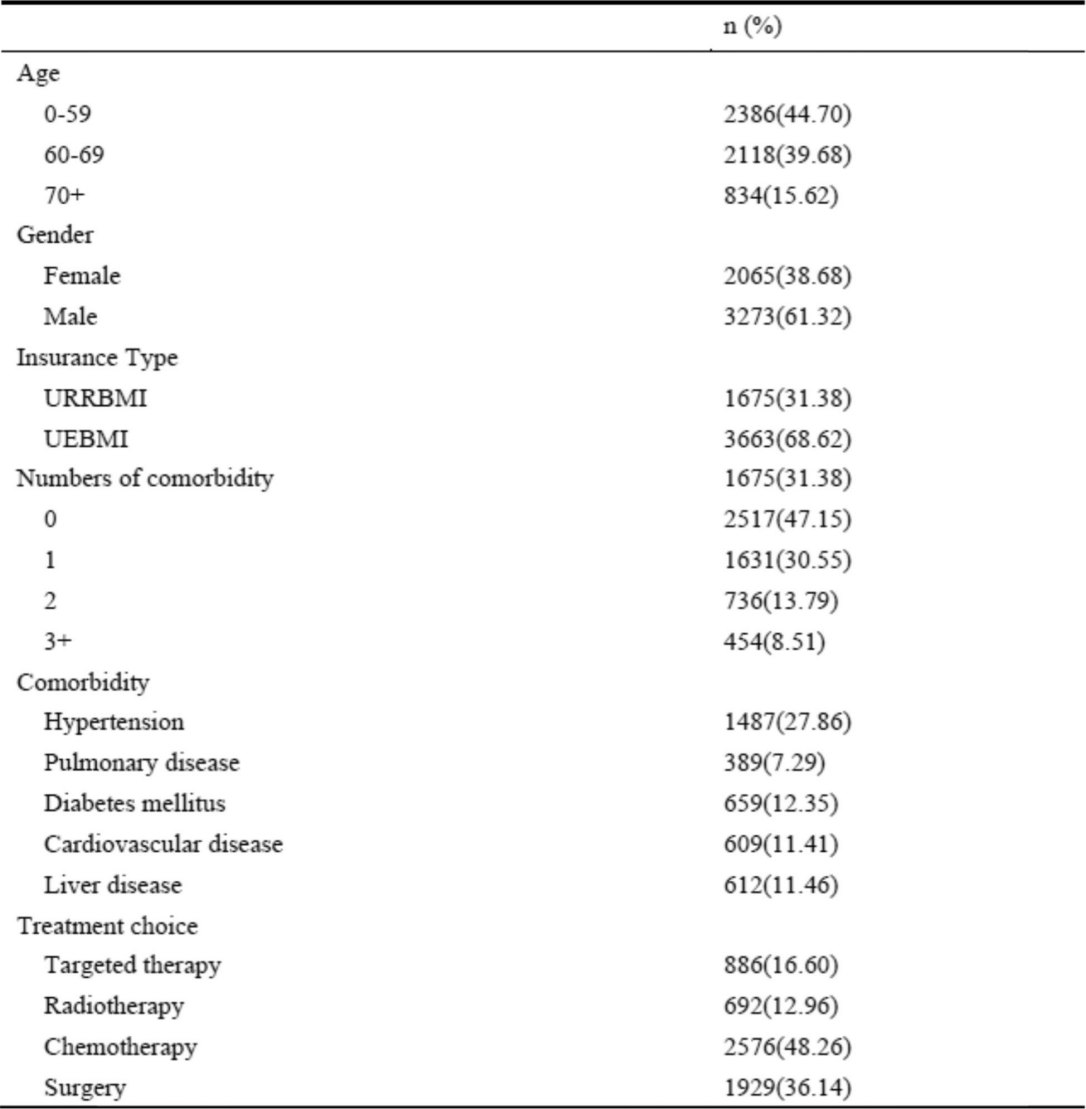
Sample characteristics (*n* = 5338)^1^

^1^Second part analysis: Hospital Information System database in BCH

*URRBMI* Urban and Rural Residents’ Basic Medical Insurance, *UEBMI* Urban Employees’ Basic Medical insurance

### Comorbidity of lung cancer patients in urban China

Table 1 presents the prevalence and the main types of comorbid conditions among lung cancer patients in the first part analysis. 31.3% of the sample had at least one comorbid conditions, and the proportion of patients having one, two and three or more comorbidities was 21.2 7.9 and 2.2%, respectively. Other malignancy, hypertension, pulmonary disease, diabetes mellitus, cardiovascular disease and liver disease were the most prevalent comorbidities in patients, and the prevalence was 7.5, 4.7, 3.8, 2.3, 2.1 and 1.9%, respectively.

Table 2 presents the prevalence and the main types of comorbidities among lung cancer patients in the second part analysis. 52.8% of the sample had at least one comorbid conditions, and the proportion of patients having one, two and three or more comorbidities was 30.6, 13.8 and 8.5%, respectively. Hypertension, diabetes mellitus, liver disease, cardiovascular disease and pulmonary disease, were the most prevalent comorbidities, and the prevalence was 27.9, 12.4, 11.5, 11.4 and 7.3%, respectively.

### Association between the presence of comorbid conditions and medical service utilization and expenditure among lung cancer patients in urban China (first part analysis)

**Fig. 1:**
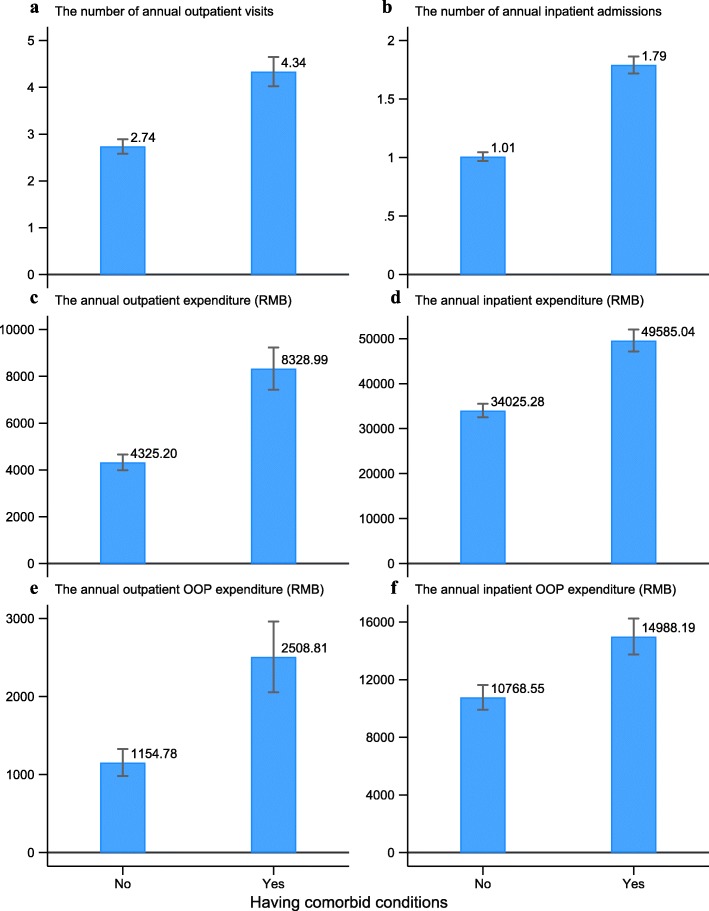
Predicted medical service utilization and expenditure with 95%CI by the presence of comorbidity among lung cancer patients (OOP, Out-of-pocket) (first part analysis: claims data)

Figure 1 displays the comparison of medical care utilization and expenditure between lung cancer patients with and without comorbidities in urban China. The predicted annual number of outpatient visits and annual inpatients admissions were 4.3 visits and 1.8 admissions, respectively, among lung cancer patients with comorbidities, which were 1.6 visits (95%CI: 1.3, 1.9) and 0.8 (95%CI: 0.7, 0.9) admissions higher than those without any comorbid conditions (Fig. 1a and b). The predicted annual outpatient and inpatient expenditure were RMB4 003.8 (95% CI: 3089.1, 4918.4) (USD635.5 (95%CI: 490.3, 780.8) and RMB15 562.7 (95%CI: 12591.4, 18,534.0) (USD2 470.3 (95CI%: 1998.6, 2941.9) higher compared to their non-comorbid counterparts (Fig. 1c and d). The predicted annual outpatient and inpatient OOP expenditure were also increased by RMB1 354.0 (95%CI: 906.2, 1801.9) (USD214.9 (95%CI: 143.8, 286.0) and RMB4 220.6 (95%CI: 2681.5, 5759.9) (USD669.9 (95%CI: 425.6, 914.3), respectively, among patients with comorbidities (Fig. 1e and Fig. 1f). The result of negative binomial regression showed statistical significance (*P* < 0.001) in all above-mentioned associations.

### Association between the number of comorbidities and medical service utilization and expenditure among lung cancer patients in urban China (first part analysis)

**Fig. 2:**
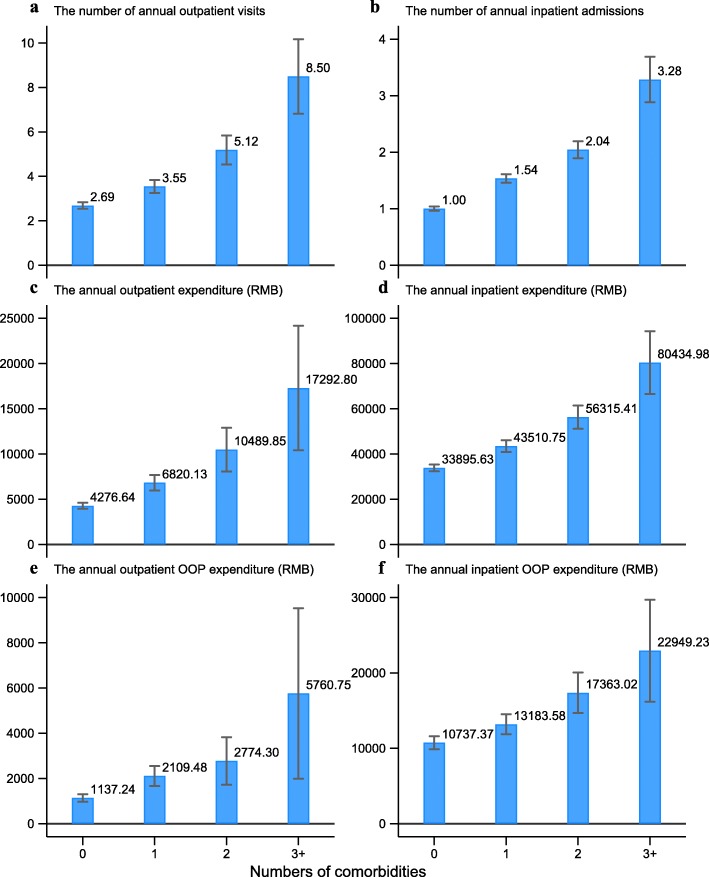
Predicted medical service utilization and expenditure with 95% CI by number of comorbidities among lung cancer patients (OOP, Out-of-pocket) (first part analysis: claims data)

Figure 2 displays the comparison of medical care utilization and expenditure among lung cancer patients from claims data by number of comorbidities. The predicted number of annual outpatient visits among lung cancer patients with 0, 1, 2, and 3 or more comorbidities were 2.7, 3.5, 5.2 and 8.5 visits, respectively (Fig. 2a). The predicted number of annual inpatients admissions increased with the number of comorbidities, with 1.0, 1.5, 2.0 and 3.3 admissions among patients with 0, 1, 2, and 3 or more comorbid conditions (Fig. 2b). The annual outpatient and inpatient expenditure were increased from RMB4 276.6 (USD678.8) and RMB33 895.6 (USD5 380.3), respectively, among patients without any comorbidities to RMB17 292.8(USD2 744.9) and RMB80 435.0 (USD12 767.5), respectively among patients with 3 or more comorbidities (Fig. 2c and d). Similarly, the annual outpatient and inpatient OOP expenditure were also increased from RMB1 137.2 (USD1 80.5) and RMB10 737.4 (USD1 704.3), respectively among patients without any comorbidities to RMB5 760.8 (USD914.4) and RMB22 949.2(USD3 642.7), respectively, among those with 3 or more comorbidities (Fig. 2e and f). The result of generalized linear model showed statistical significance in all above-mentioned associations.

### Association between six main types of comorbidities and excess medical care utilization and expenditure among lung cancer patients in urban China (first part analysis)

Table 3 displays the association of excess medical service utilization and expenditure with different types of comorbidities among lung cancer patients from claims data. In annual outpatient service, the predicted number of visits for patients with hypertension, cardiovascular disease and liver disease were significantly decreased by 0.62 (=1-exp^0.484^, *P* < 0.05), 0.65(=1-exp^0.500^, P < 0.05) and 1.80(=1-exp^1.030^, *P* < 0.001) visits, respectively compared with those without any comorbidities. And other malignancy is the only comorbidities significantly associated with increased annual outpatient expenditure. The OOP expenditure of outpatient visits were significantly increased by 78.2% (=exp^0.578^–1, *P* < 0.01) and 105.2%(= exp^0.719^–1, P < 0.05), respectively among patients with other malignancy or liver disease compared to those without any comorbidities. However, we also observed 54.8% (=1-exp^0.437^, P < 0.01) significantly decrease in the OOP expenditure among patients with hypertension. In annual inpatient service utilization, the predicted number of inpatient admissions among patients with other malignancy, hypertension, pulmonary disease, diabetes mellitus, cardiovascular disease and liver disease were 0.362, 0.476, 0.175, 0.292, 0.425 and 0.420 admissions significantly higher, respectively, compared to their non-comorbid counterparts. And all six types of comorbid conditions are significantly associated with increased annual inpatient expenditure. Similarly, all types of comorbid conditions are significantly associated with increased annual inpatient OOP expenditure except for those with pulmonary disease.

**Table 3:**
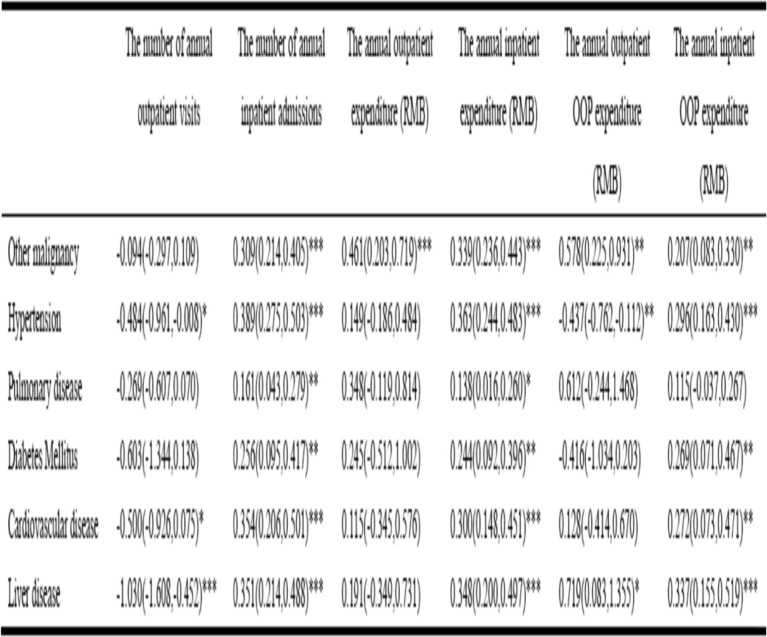
Adjusted association between six main types of comorbid conditions and medical service utilization and expenditure: Coefficient & 95%CI^1^

^1^First part analysis: claims data

All models were adjusted for gender, age group, insurance type, city and year. *** *p* < 0.001, ** *p* < 0.01, * *p* < 0.05. OOP, Out-of-pocket

### Association between comorbidity and different treatment choice among lung cancer patients in China (second part analysis)

Table 4 displays the association between comorbidity and different treatment choice among lung cancer patients in China based on data from Hospital Information System (HIS) Database in the Beijing Cancer Hospital (BCH). Having comorbidity was associated with increased possibility of choosing radiotherapy (OR: 1.208, 95%CI:1.012–1.441) and chemotherapy (1.363, 1.196–1.554), and decreased possibility of choosing surgery (0.850, 0.730–0.989).

**Table 4:**
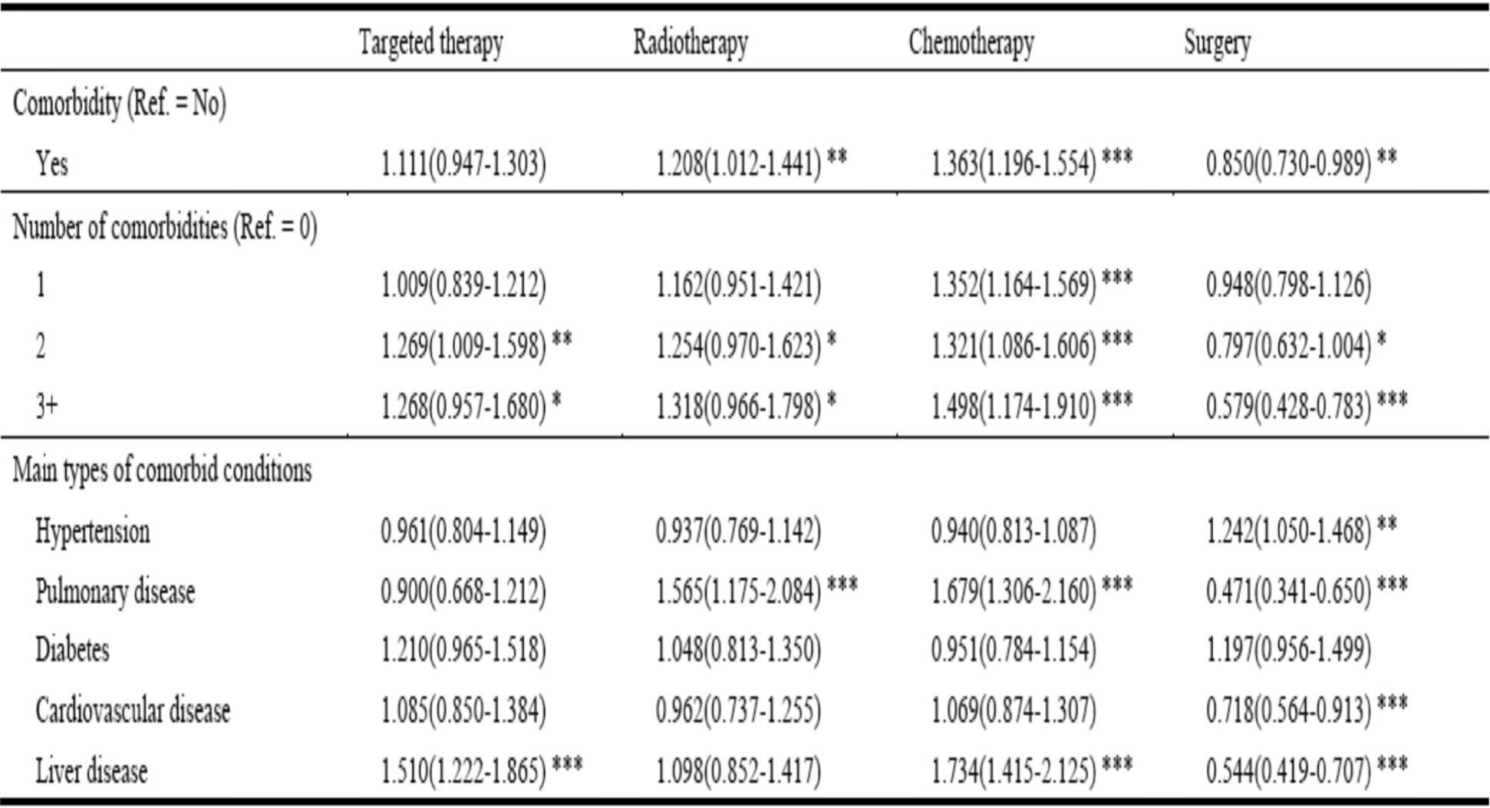
Adjusted association between comorbid conditions and treatment choice: Odds Ratio & 95%CI^1^

^1^Second part analysis: Hospital Information System Database

All models were adjusted for gender, age group, insurance type and lung cancer stage. OR and 95% CI were reported, and 95% CI in parentheses. *** *p* < 0.001, ** *p* < 0.01, * *p* < 0.05

Similar result also found in the association between number of comorbidities and different treatment choices. The possibility of choosing chemotherapy among patients with 1, 2, and 3+ comorbidities was significantly increased by 35.2% (1.352, 1.164–1.569), 32.1%(1.321,1.086–1.606) and 49.8% (1.498, 1.174–1.910) compared with their counterparts without comorbidity. Similar pattern was also observed for targeted therapy among patients with 2, or 3+ comorbidities. The possibility of choosing radiotherapy significantly increased, and the possibility of choosing surgery significantly decreased with the number of comorbidities among lung cancer patients.

Table 4 also showed the result of the association between different types of comorbidities and different treatment choices. Having pulmonary disease was associated with increased possibility of choosing radiotherapy (1.565, 1.175–2.084) and chemotherapy (1.679, 1.306–2.160), and decreased possibility of choosing surgery (0.471, 0.341–0.650). And cardiovascular disease was associated with decreased possibility of choosing surgery (0.718, 0.564–0.913). Similarly, having liver disease was associated with increased possibility of choosing targeted therapy (1.510, 1.222–1.865) and chemotherapy (1.734, 1.415–2.125), and decreased possibility of choosing surgery (0.544, 0.419–0.707).

## Discussion

Based on medical insurance claims data, this study firstly presents the association of medical care service utilization, expenditure and treatment choice with comorbidity in lung cancer patients in urban China. Overall, more than one third of lung cancer patients have comorbidity. The Health care utilization and cost was significantly increased among patients with comorbidity and it increases with increasing number of chronic conditions. In addition, our study showed that comorbidity was associated with different treatment choices among lung cancer patients. And there are variations of outpatient and inpatient service utilization and expenditure, as well as treatment choice in individuals with different types of comorbid conditions.

Our result showed that 31.3% of lung cancer patients in China have comorbidities, among which the most commonly identified chronic diseases were other malignancy, hypertension, pulmonary disease, diabetes mellitus, cardiovascular diseases and liver disease. Although the prevalence of comorbidity in urban China was lower than that in developed countries, which ranges from 43.3% in Sweden [7] to 87.3% in Scotland [8], the younger median age of Chinese lung cancer patients (65) in our study compare to > = 68 in study from developed countries) and the heterogeneity of data source may contribute to the difference [14]. Besides advanced age as the key predictor of comorbidity [15], it has also been suggested that cigarette smoking contributed to the risk of concomitant chronic respiratory diseases and cardiovascular diseases among lung cancer patients [14], which may be the case for China, as there are 350 million smokers and they consumed approximately 30% of world tobacco every year [16].

The most important finding is that comorbidity was associated with higher service utilization and expenditure for both outpatient visits and inpatient admissions among lung cancer patients in urban China. According to the estimation, comorbidity in lung cancer patients incurred an increase of more than 50% in the annual number of outpatient visits, an increase of 92.5% in outpatient expenditure, and an increase of RMB15562 (USD2 470.2) in inpatient medical cost, which is amount to half of annual personal disposable income for Chinese urban residents in 2015 [17]. Our result indicates that comorbidity among lung cancer patients add substantial burden to medical insurance system in China. Though there is a scarce of existing literature on the association between comorbidity and medical service utilization and cost in lung cancer patients, similar results have been found in studies of comorbidity on patients with other diseases. For example, one study in Canada [18] suggested that use of all health services and medical cost was increased with comorbidity among older adults with stroke. An association between the presence of comorbidities and all-cause hospitalizations and health care costs was also reported among chronic obstructive pulmonary disease (COPD) patients [19]. Patients with more than one type of chronic diseases would naturally require more care and additional medical resources, and the impact of comorbidity on the treatment and performance of lung cancer patients has been demonstrated by a series of studies [20, 21]. Management decisions with comorbidity always requires multidisciplinary consultation [7], and post-operative complications was found to be associated with comorbidities among lung cancer patients [22], both of which may result in increased outpatient visit, prolonged hospitalization and the rising medical expenditure.

The results of the increased use of outpatient and inpatient service with the increasing number of comorbidity in our study are in line with existing similar research [23–25]. In a Dutch study [26], the mean number of annual medical contacts in patients with multi-morbidity patients was 18.3, significantly higher compared to those with only one (11.7) or none chronic conditions (6.1). One study from Germany [25] showed that each additional chronic disease was associated with an increase of 2.3 medical contacts. And the increase in medical costs with increasing number of chronic condition was also reported by a Swiss study [27], from which each additional comorbidity was associated with increased total costs of 2383 USD per year. However, comparing with the exponentially rise conclude from a systematic review of several studies [24], the increase in both outpatient and inpatient expenditure with the number of comorbidity among lung cancer patients was only moderate in our result, which may be explained by the fact that lung cancer was considered as index disease in the extraction of information on medical service utilization and expenditure and the cost for other chronic disease may be underestimated.

The association between different types of comorbidities and increased medical service utilization and expenditure among lung cancer patients was observed for inpatient admissions. However, the result suggested that hypertension, cardiovascular diseases and liver diseases were each associated with decreased number of outpatient visits. This may be explained by the trade-off between outpatient and inpatient visits since hypertension, cardiovascular diseases and liver diseases were the three comorbidities with the largest increase of inpatient admission numbers. And the greater hospitalization for comorbid hypertension and cardiovascular diseases was also reported from prior research [19] on COPD patients. Nevertheless, given a limited explanation for this issue, further in-depth investigation is required to explore the causation.

It is also worth to mention the association between comorbidity and different treatment choice. Our result showed that having comorbidity, increased number of comorbid conditions, and having certain types of comorbidity (i.e. pulmonary disease or liver disease) was associated with increased possibility of receiving conservative treatment like chemotherapy, and decreased possibility of receiving curative treatment like surgery. Similar phenomenon has been reported across different cancer sites and health settings [28]. The possibility that the treatment choice was depending on comorbid conditions should be ruled out, because comorbidity was still absent in the latest edition of Chinese Medical Association guidelines for clinical diagnosis and treatment of lung cancer [29], in which the selection criteria of surgery, chemotherapy, radiotherapy and targeted therapy were mainly based on lung cancer stage, performance status and genetic test. Cancer patients with comorbid conditions are generally less likely to receive surgery or other curative treatment. The increased risk of treatment toxicity, side effects and post-operative complications associated with comorbidity or the concern that the life expectancy of patients with comorbidity is insufficient to justify the use of potentially toxic therapy may prevent clinicians from aggressive treatment choices [30]. Additionally, there was a lack of high-level evidence on the effect of cancer therapies among patients with comorbidity since most of randomized controlled trials always exclude those with concomitant conditions. The insufficient evidence further restrains clinicians’ choice and lead them turn to conservative treatment [28]. Our finding underlines the necessity of research on curative therapy among patients with comorbidity and the development of treatment decision aids incorporated with the impact of comorbidity on survival and quality of life for clinicians, and also highlights the potential challenges of comorbidity in the management of lung cancer treatment in China.

### Implications

The current study offers a comprehensive estimation of the association between comorbidity and lung cancer patients’ treatment choice, medical service utilization and expenditure, in a predominantly urban medical insurance population in China. Together with the assessment of specific comorbidities, it provides information for China medical insurance management and medical professionals to develop enhanced risk assessment protocols and tailored therapeutic interventions for lung cancer patients with comorbidity. And the overview of comorbidity situation, as well as the estimation of extra cost in lung cancer patients further highlights the importance of tobacco control promotion among Chinese population. In addition, this study quantifies the extra burden of comorbidity on different types of medical service utilization and expenditure, which allows government to estimate the budget and resource allocation, and to enhance modeling of potential return on prevention investment.

### Limitations

The current study has several limitations. First, we are unable to further investigate the specific reasons for the extra service utilization and costs due to a lack of related clinical information of each outpatient visit and inpatient admission in the data source. Secondly, lung cancer was considered as the index disease in collecting information on medical service utilization and expenditure, so that the cost for other chronic conditions may be underestimated. And the exclusion of the cases that consider lung cancer as the comorbidity and other diseases as index disease may also result in potential bias. Thirdly, the heterogeneity between two different data set that employed in our study should be noted, and the extrapolation from the second part analysis should be cautious because the sample may not be representative of the lung cancer patients in China. Despite of above limitation, the main strength of this study is using China urban basic insurance claims data and hospital information system data, firstly examined the impact of comorbidity on medical utilization and expenditure, as well as treatment choice among lung cancer patient, providing a comprehensive understanding and raising the attention of comorbidity among lung cancer patients in developing countries.

## Conclusion

The presence of comorbidities among lung cancer patients restricts the potential treatment choices and pose an extra substantial health care burden with significantly higher medical service utilization and expenditure compared to lung patients without comorbidity. These findings provide information for relevant authorities to identify patients at greater needs of medical services by assessing comorbidity profiles in effort to target lung cancer care management resources. Insights from this study may also contribute to the development of more comprehensive disease management programs to improve patients’ quality of life, in the meantime of cost management.

## Data Availability

The data were provided by China Health Insurance Research Association and Beijing University Cancer Hospital. These are third party data. Authors in this study have the right to use this dataset, but not the right to share and distribute. A de-identified minimal dataset of the quantitative data is available upon request to researchers who meet the criteria for confidential information, by sending a request to phe@pku.edu.cn.

## Abbreviations

UEBMI: Urban Employees’ Basic Medical Insurance;
URBMI: Urban Residents’ Basic Medical Insurance;
HIS: Hospital Information System;
BCH: Beijing Cancer Hospital;
GLM: Generalized linear model;
DALY: Disability adjusted life years;
CHIRA: China Health Insurance Research Association;
ICD: International Statistical Classification of Diseases;
OOP: Out-of-pocket;
TNM: Tumor-node-metastasis
